# Selection of recombinant MVA by rescue of the essential D4R gene

**DOI:** 10.1186/1743-422X-8-529

**Published:** 2011-12-12

**Authors:** Patricia S Ricci, Birgit Schäfer, Thomas R Kreil, Falko G Falkner, Georg W Holzer

**Affiliations:** 1 Baxter BioScience, Biomedical Research Center, Uferstrasse 15, 2304 Orth an der Donau, Austria; 2 Baxter BioScience, 1221 Vienna, Austria

**Keywords:** Recombinant vaccines, Uracil-DNA glycosylase, Vaccinia virus, Cell Line, Transformed, Defective viruses/genetics, Defective viruses/growth & development

## Abstract

Modified vaccinia virus Ankara (MVA) has become a promising vaccine vector due to its immunogenicity and its proven safety in humans. As a general approach for stringent and rapid selection of recombinant MVA, we assessed marker rescue of the essential viral D4R gene in an engineered deletion mutant that is fully replication defective in wild-type cells. Recombinant, replicating virus was obtained by re-introduction of the deleted viral gene as a dominant selection marker into the deletion mutant.

## Introduction

Modified vaccinia virus Ankara (MVA) was developed as a safer smallpox vaccine during the smallpox eradication campaign [1]. For this purpose, vaccinia strain Ankara was passaged more than 500 times in primary chicken embryo fibroblast cells (CEF). Replication of MVA is almost completely restricted to avian cells, and the virus is strongly attenuated *in vivo*. The excellent safety record of this strain was established in vaccination campaigns in the 1970's, when over 120,000 humans including immune suppressed individuals were immunized [2,3]. Safety of MVA as a smallpox vaccine has also been addressed by others in clinical studies [4-6].

The attenuated phenotype is the consequence of multiple changes in the viral genome. Six major deletions and numerous other mutations including small deletions, point mutations and insertions relative to the parental strain were mapped [7,8]. MVA has been intensively studied as a vector for use in recombinant live vaccines [9]. MVA-based live vaccines have already been under evaluation in numerous clinical trials, recently for instance for HIV [10,11], influenza [12], and against smallpox [5]. Safety was also demonstrated with a recombinant MVA-based cancer vaccine (TroVax) in clinical studies [13].

Recombinant MVA are usually generated by homologous recombination techniques [14,15], followed by repeated rounds of plaque purification under selective conditions. Methods for clonal selection have been established previously for vaccinia virus in general, including dominant E. coli gpt marker selection [16] and lacZ color screening [17], and are equally used for work with MVA. Other techniques for the isolation of recombinant vaccinia virus (VV), such as tk^- ^selection, cannot be applied to MVA due to its restriction to a few cell types. Specifically for MVA, certain host range selection methods have been developed that make use of the genetic basis of the growth restrictions of MVA. For example, introduction of the VV gene K1L as a marker which is naturally deleted in MVA, extends the host range to rabbit kidney cells [18]. However, there might be regulatory concerns that selection markers can change the attenuated phenotype of MVA thereby impairing the well-established safety profile of MVA. For instance, the VV K1L gene encodes viral functions that impair important anti-viral defense mechanisms of the infected host. The K1L gene product suppresses activation of the transcription factor NF-κB [19]. More recently, it was shown that K1L -alongside with C7L- is also involved in preventing RNA-activated protein kinase (PNK) -dependent and -independent shut-off of protein synthesis by the infected host [20]. In MVA, the lack of a functional K1L gene enables early-phase PNK-mediated host responses possibly contributing to both the impairment of viral replication and the stimulation of the immune response [21].

Generally, plaque purification procedures are more difficult with MVA compared to replicating vaccinia strains, because MVA work is limited to a narrow range of cell substrates. Classically, MVA is grown and purified on primary CEF. Plaque formation on these cells is not as clear as with most permanent cell lines, and the work depends on cell supply of constant quality. To compensate for these shortcomings, additional detection methods such as immunostaining have been applied for MVA plaque isolation on CEF [22].

An elegant and stringent selection approach is the rescue of virus with wild-type (wt) -like growth properties by re-introduction of a previously deleted viral function. For MVA, deletion of the non-essential genes E3L and F13L, respectively [23,24] resulted in viruses of altered plaque properties or narrowed host range, allowing the selection of recombinants after re-introduction of these genes and rescue of the wild-type phenotype.

While the latter selection methods are based on non-essential host range genes, we now attempted a rescue selection procedure by use of a parental MVA that lacks an essential function and that is entirely growth incompetent on wild-type cells. Deletion of the essential gene D4R from vaccinia virus and complementation of the deleted viral function by an engineered complementing cell line has been described earlier [25]. The vaccinia virus gene D4R encodes the protein uracil DNA glycosylase, which catalyzes the removal of uracil residues from double-stranded DNA, and which is an indispensable component of the vaccinia DNA polymerase complex [26]. Recently, knock-out of the D4R gene has been applied to MVA with the purpose of reduced vector antigen expression in recombinant vaccines [27]. Growth of the knock-out virus requires a permanent cell-line that may be engineered to complement the viral function. For MVA which is largely restricted to avian cells, the avian cell line DF-1 was found to be appropriate for this task. The cell line DF-1 had been created after spontaneous immortalization of CEF and was initially investigated for the work with avian retroviruses [28]. Because D4R-based dominant host range selection was found to provide a powerful selection method for replicating vaccinia recombinants [29], it was now attempted to adopt this technique also to MVA.

## Materials and methods

### Cell lines and viruses

The African green monkey cell line Vero (ATCC CCL-81), and the chicken embryo fibroblast cell line DF-1 (ATCC CCL-12203) were grown in DMEM (Biochrom) containing 5% of fetal calf serum (PAA), non-essential amino acids (NEAA) and Pen/Strep (Lonza). Primary chicken embryo cells (CEF) were isolated from 12 day old chicken embryos and grown in Med-199 (Gibco) containing 5% of fetal calf serum, Pen/Strep and NEAA.

Construction of the D4R defective vaccinia virus Lister (dVV-L) is described elsewhere [30]. Modified vaccinia virus Ankara (Isolate MVA74 LVD6) was obtained from Dr. Bernard Moss, National Institute of Allergy and Infectious Diseases. This MVA isolate originates from three rounds of plaque purifications of MVA passage 572 [1] and was frozen prior to the emergence of bovine spongiform encephalopathy. The virus rMVA-YF/d3 that carries the YFV prME cassette in the genomic del3 region is described elsewhere [31].

### Retroviral transduction of DF-1 cells

The D4R gene was introduced into DF-1 cells by transduction with a retroviral vector that had been derived from the commercially available vector pLXSN, Invitrogen [32]. Retroviral vector particles were generated in Vero cells by simultaneous transfection with the packaging plasmids pGAGPOL-gpt [33] and pALF (both kindly provided by W. Günzburg, University of Veterinary Medicine Vienna), and the vector plasmid pLXSN-D4R [34]. The plasmids (7 μg each) were transfected into subconfluent Vero cells by calcium phosphate transfection as described before [35]. After 2 days, the supernatant was collected, supplemented with 4 μg/ml polybrene and filtered through 0.45 μm filters (VWR). The retroviral vector containing supernatant was diluted 1:10 and used to infect DF-1 cells of passage 31. After 4 days, cells were split into medium containing 100 μg/ml G418 (Invitrogen). Media changes were made every 4 days until cell foci could be picked and expanded under continued selective pressure. In passage 38, a master cell bank of cDF-1 was frozen, of which one aliquot was thawed and expanded for a working cell bank. The working cell bank corresponds to passage 41 of the parental DF-1 line.

### Construction of plasmids

#### pDM-Zgpt

The plasmid pDM-Zgpt contains the lacZ/gpt double marker cassette flanked by genomic sequences of MVA to drive homlogous recombination into the viral D4R locus. First, the MVA sequences, that flank the D4R gene at either side, were amplified by PCR, using MVA genomic DNA as the template. The left flanking region of the D4R gene was amplified using the forward primer 5'-CAT TGT TAA CTG TGA GCT ACT GTA GGT G-3' and the reverse primer 5'-CTC GAG GTC GAC AAG CTT CCA TGG TTA TAT CAA ATT AGA TAC C-3'. For cloning purposes, a HpaI restriction site (restriction sites underlined) was introduced into the forward primer. The reverse primer contained XhoI, SalI, HindIII and NcoI as a future multiple cloning site. The MVA D4R right flank was generated by PCR with the forward primer 5'-GTC GAC CTC GAG GGC GCC GCG GCC GCT GCT TTA GTG AAA TTT TAA C-3' containing the restriction sites SalI, XhoI, EheI, NotI, and the reverse primer 5'-CGT AAG CGG AAG AGC ACT ATT GTT GTT CAT ATC CAC G-3'. The PCR products were sequentially cloned into pPCR-ScriptAmpSK(+) (Stratagene). In the resulting construct pDM, the MVA D4R flanks (left flank: 836 bp; right flank: 685 bp) frame a multiple cloning site. Next, the lacZ/gpt double marker cassette from pDW-2 [29] was cloned via HindIII/SalI restriction into pDM to generate pDM-Zgpt.

#### pDM-D4R

The MVA D4R gene was amplified by PCR from MVA genomic DNA with the forward primer 5'-AGG CGT TTG TAT TCG CTT GG-3', and the reverse primer 5'-ACA CCA TGG GCT AGC TCG CGA TTA ATA AAT AAA CCC TTG AGC C-3'. The forward primer binds to the genomic MVA DNA upstream of an Eco105I site that is located 27 bp outside of the the D4R gene. The reverse primer contained the restriction sites NcoI, NheI and NruI for a MCS downstream of the D4R gene. Using the restriction sites Eco105I and NcoI, the PCR product was cloned into pDM resulting in pDM-D4R.

#### pDM-D4R-YF

To construct the plasmid for integration of the YFV prME into the D4R/D5R locus, the yellow fever prME gene cassette was excised from plasmid pd3-YFprMEco and cloned into pDM-D4R, using the restriction sites NheI and SalI. The resulting plasmid pDM-D4R-YF contains the YFV prME expression cassette downstream of the D4R gene [31].

### Generation of viruses

Recombinant virus was generated by homologous recombination [15]. Confluent monolayers of the indicated cell types were infected/transfected according to the procedures essentially described before [34].

#### dMVA-ZG

Infection/transfection with MVA-wt and the plasmid pDM-Zgpt was done in CEF. Recombinant MVA was selected by seven rounds of plaque purification with gpt selection [16] and lacZ screening [17] in the D4R complementing cell line cDF-1. A plaque isolate that did not show any growth on wt DF-1 cells was expanded in cDF-1 and a purified virus stock was prepared as previously described [36]. The resulting defective MVA was termed dMVA-ZG.

#### rMVA-YF/D4

Wild type DF-1 cells were infected with dMVA-ZG, and the plasmid pDM-D4R-YF was used for transfection. Plaque purification and amplification were carried out in wild type DF-1 cells.

### Characterization of defective MVA by PCR

PCR was performed to confirm the expected genomic structure of the defective virus dMVA-ZG and to test for contamination with residual wild-type MVA. For this purpose, sucrose cushion purified dMVA-ZG stock was used. For spike controls, 1%, 0.1%, 0.01% and 0.001% MVA-wt were added to dMVA-ZG samples. After digestion with 0.5 μg/μl proteinase K (Sigma) for 3 hrs at 56°C and 2 hrs at 95°C, 1 μl samples corresponding to 1 × 10^5 ^pfu were subjected to PCR.

The reaction was carried out with the primers 5'-ACC TTC CAA CTG TGG ATA CTC TG-3' and 5'-TCG AAT GAA ATA AAC CCT GGT-3' resulting in a PCR signal of 2430 bp with MVA-wt, and of 5624 bp with dMVA-ZG as a template.

### Growth experiments with MVA and vaccinia virus

Confluent cells cultivated in 6-well plates were infected with 5 × 10^4 ^tissue culture infectious doses 50% (TCID_50_) of the respective MVA or vaccinia virus, followed by media change after 1 h. Cells were kept in a CO_2 _incubator at 37°C. At the respective time points post infection, cells were scraped into the medium and disrupted by three cycles of freeze/thaw and ultrasonication. The virus titer was determined by TCID_50 _assay, and the output/input ratio was calculated. MVA was grown and titrated on the cell line DF-1, and dMVA-ZG on the complementing cell line cDF-1.

### Determination of infectious titers

Infectious titers were assayed by TCID_50 _assay. Serial ten-fold dilutions of specimen were applied to CEF in 96-well plates, and virus induced cytopathic effect was evaluated by light microscopy after 5-7 days of incubation at 37°C in a CO_2 _atmosphere. Viral titers were calculated according to the formula of Spearman and Kaerber [37] and were expressed as TCID_50_/ml. One TCID_50_/ml corresponds to about 0.5 plaque forming unit (pfu), as determined in a series of parallel measurements.

### Double immunostaining

Plaques of MVA that express YFV prME antigen were identified by double immunostaining.

cDF-1 cells were infected with recombinant YF prME-expressing MVA for a period of 4 days, and were then fixed with methanol/acetone (1:1). To detect plaques of YFV prME expressers, a guinea pig antiserum against YFV-17D (Lot # 070824/T4, Baxter) was used. Goat anti-guinea pig horseradish peroxidase-conjugated IgG (Jackson ImmunoResearch Laboratories, Inc.) was used as a secondary antibody. Plaques were visualized with diaminobenzidine (DAB) solutions including nickel (Vector Laboratories), resulting in black plaques. In a subsequent round, plaques were additionally stained for vaccinia antigens, using a polyclonal rabbit anti-vaccinia virus serum (Lot no. VVSKP26012006, Baxter). In this case, the secondary antibody was a goat anti-rabbit peroxidase-conjugated IgG (Jackson Inc), and staining was done with DAB solution without nickel, resulting in brown plaques, essentially as described earlier [38].

### Growth assay of defective MVA for replicating contaminants

To detect replication competent virus in defective virus stocks, ten 175 cm^2 ^cell culture flasks of confluent DF-1 cells were infected with a total of 2 × 10^6 ^pfu (moi = 0.05) of dMVA-ZG. In parallel cell culture flasks were infected with 100 pfu wild-type MVA or with a mixture of 2 × 10^6 ^pfu dMVA-ZG and 100 pfu wild-type MVA to control the sensitivity of detection. Uninfected DF-1 cells served as a negative control. Five days post infection plaques of residual replication competent virus were visualized by crystal violet staining.

### Western blotting

Whole cell lysates were prepared from 5 × 10^6 ^infected cells by three cycles of freezing/thawing, followed by ultrasonication and denaturation in protein loading buffer (Fermentas). Aliquots corresponding to 1 × 10^5 ^cells were resolved by 12% polyacrylamide gel electrophoresis. Blotting was performed essentially as described previously [39]. For detection, a polyclonal guinea pig antiserum (Lot # 070824/T4, Baxter) against YFV-17D, and anti-guinea pig alkaline phosphatase-conjugated immunoglobulin (Sigma) as the second antibody, were used.

### Isolation of genomic DNA and real-time PCR

Recombinant virus plaques were picked and suspended in 500 μl DMEM, and used to infect cDF-1 cells in 6-well plates to amplify the recombinant virus and potential residual parental dMVA in the stocks. As controls, cells were infected in parallel with the parental dMVA-ZG, wild-type MVA and rMVA-YF/del3. After 5 days, infected cells were harvested, and genomic DNA was isolated using DNeasy kit (Quiagen Inc.). Aliquots corresponding to 0.4% of the 1 ml harvest from a 6-well were used as template in quantitative PCR reactions. Real-time PCR was performed with 2 μl of genomic template DNA in a total volume of 20 μl using Taqman gene expression master mix (Applied BioSystem), and primers and probes at a final concentration of 900 nM and 200 nM, respectively, using a StepOnePlus real time PCR system (ABI, 4376598).

For amplification of the individual genomic targets, the following primer-probe sets were used:

YFV prME-specific: FWD (5'-TGA TGC AGG TCA AAG TGT CTA AGG-3'), RWD (5'-TGT TGA TGG CGG CTG TCA-3'); and Probe (5'-6FAM-CTG CCG GAT CCC CGT-MGB-3').

LacZ-specific: FWD (5'-ATT CAG GCT GCG CAA CTG TT-3'), RWD (5'-CAG CAC ATC CCC CTT TCG-3'), and Probe (5'-6FAMAAG GGC GAT CGG TGC G-MGB-3').

MVA 173R gene-specific: FWD (5'-GCA ACG GCG AAA CAA AAT ATT T-3'), RWD (5'-ATT AGG ACA CGT AAC AGT ATC ATT CCA-3'), and Probe (5'-6FAM-TTG CGA AGA AAA AAA TGG AA-MGB-3').

Specific for plasmid DNA (Amp^r ^gene): FWD (5'-CCA ACG ATC AAG GCG AGT TAC-3'), qPCRVVamp-RWD (5'-CCG AAG GAG CTA ACC GCT TT-3'), and Probe (5'-6FAM-TGA TCC CCC ATG TTG TG-MGB-3').

To generate standard curves for determination of genomic equivalents, plasmids containing Amp^r ^-, lacZ and 173R sequences and a plasmid containing the YFV prME gene, were used in ten-fold dilutions ranging from 1 × 10^8 ^to 1 × 10^3 ^DNA copies.

## Results and discussion

### Generation of the D4R complementing cell line cDF-1

Traditionally, MVA is propagated in primary CEF. In order to propagate the D4R-deleted MVA that should serve as the parental strain for the intended selection procedure, a stably D4R-expressing cell line was required. Ideally, the cell line should be non-tumorigenic and of avian origin to allow the growth of MVA. For technical reasons, formation of clear MVA plaques is a desirable general characteristic of the cell line of choice. To evaluate the cell line DF-1 for the intended selection of recombinant replicating MVA, we assessed it for propagation of MVA using primary CEF as a reference. In DF-1, a maximal MVA titer (about 7.4 log10 TCID_50_) was reached as early as day 1. In plaque assays, MVA formed confined, circular plaques on DF-1 cells, making this cell line useful for the intended plaque purification of defective MVA.

The D4R complementing DF-1 based cell line was generated by retroviral transduction analogous to a Vero-based complementing cell line described previously [34]. We used the D4R-defective vaccinia virus Lister dVV-L [30] to screen for the cell clone that complemented most efficiently the lacking viral function. The DF-1 cell clone #4A allowed amplification of the defective vaccinia virus to the highest titers (data not shown), and was therefore further expanded to obtain the cell line cDF-1.

### Generation of a non-replicating parental virus

**Figure 1:**
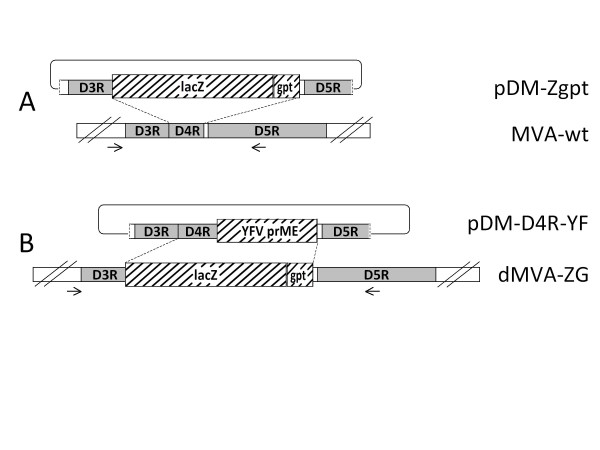
**Outline of the D4R-dominant host range selection system**. **a**. Schematic representation of the plasmid pDM-Zgpt for the construction of the D4R-deleted dMVA-ZG, and the corresponding insertion locus in the genome of MVA. **b**. Plasmid pDM-D4R-YF and the defective parental virus dMVA-ZG. MVA sequences are represented by boxes. The MVA genes D3R, D4R, D5R are marked grey. Foreign genes cassettes inserted into MVA are symbolized by hatched boxes. Arrows indicate the binding sites of the PCR primers that were used for confirmation of the structure and purity of dMVA-ZG.

**Figure 2:**
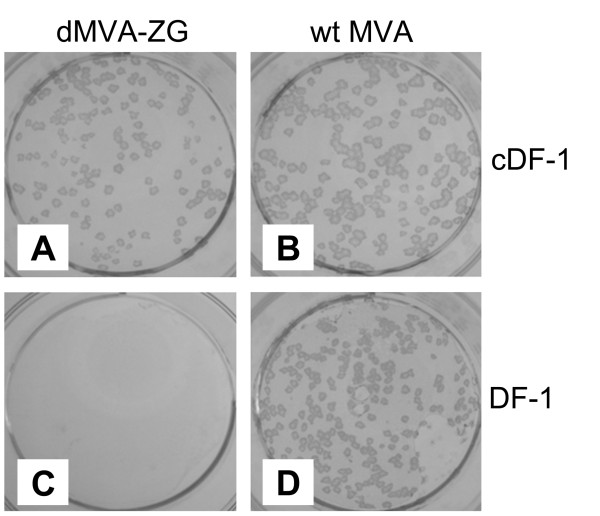
**Plaque formation of dMVA-ZG and wild-type MVA in avian DF-1 cells**. cDF-1 (**a**, **b**) and wild-type DF-1 (**c**, **d**) cells were infected with 100 pfu/well of dMVA-ZG (**a**, **c**) or MVA-wt (**b**, **d**). Four days post infection, immunostaining was performed with anti-vaccinia virus antibodies. Photographs show immune-stained plaques.

**Figure 3:**
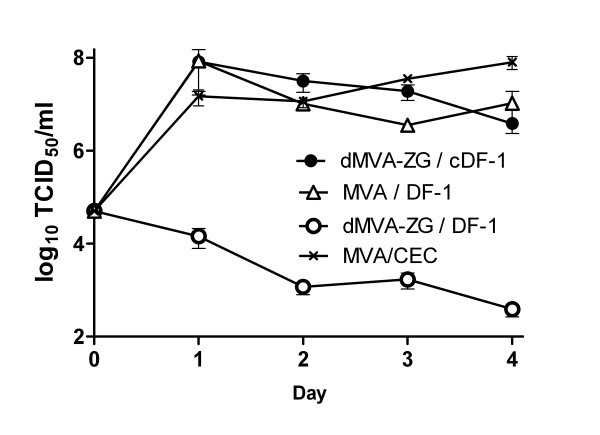
**Growth kinetics of MVA and dMVA-ZG**. cDF-1 cells were infected with the defective virus dMVA-ZG at moi = 0.05. For comparison, infections of DF-1 cells dMVA-ZG and with wild-type MVA were performed in parallel. Cells were harvested at the indicated time points and the viral titers were determined by TCID_50 _titration. Mean values ± SEM are shown (*n *= 6).

In order to knock out the essential vaccinia gene D4R, and to prepare a growth defective MVA, a recombination plasmid (pDM-Zgpt) was constructed that contains the MVA genomic regions that naturally flank the MVA D4R gene with the D4R sequences being deleted. The D4R gene and its natural promoter were replaced by a marker cassette encoding the *E. coli *lacZ and gpt genes (Figure 1a). After infection with MVA and transfection with the plasmid pDM-Zgpt, the intended recombinant virus dMVA-ZG was isolated by repeated rounds of plaque purification under gpt selection combined with blue plaque screening in the complementing cell line cDF-1. The virus dMVA-ZG did not form plaques on wt DF-1 cells (Figure 2c), while it readily formed plaques on the complementing cell line cDF-1 (Figure 2a). Plaque formation by dMVA-ZG was also absent in CEF (not shown). As expected, the MVA-wt strain grew both on cDF-1 and DF-1 cells (Figure 2b, d). On cDF-1 cells, the defective virus propagated similar to MVA-wt, while in the absence of complementation, titers decreased by about 100-fold within 4 days, indicating that the virus was completely growth negative in wt cells (Figure 3).

### The defective virus dMVA-ZG is free of MVA-wt

The absence of replicating contaminants from the dMVA-ZG stock was of special interest because the reconstitution of growth should serve as the key selection criterion for further recombinants in the dominant host range selection procedure. To phenotypically verify the absence of replicating MVA, a growth assay on DF-1 was performed that was capable to detect 1 pfu replicating MVA in 2 × 10^7 ^pfu of dMVA-ZG. No plaques were detectable after a 5 days incubation further demonstrating the purity of the preparation.

**Figure 4:**
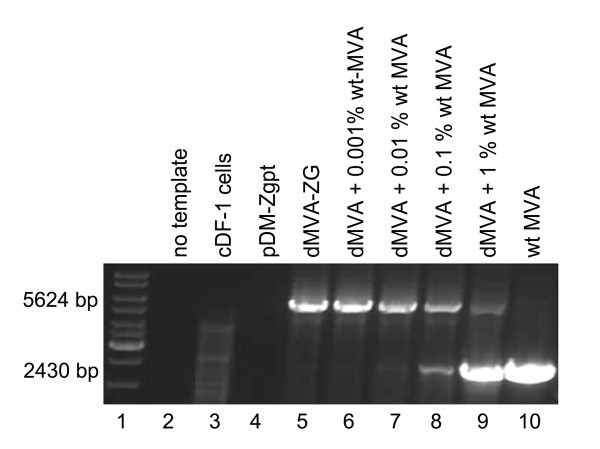
**PCR analysis of the D4R deletion in the defective virus dMVA-ZG**. Genomic DNA of dMVA-ZG was subjected to PCR amplification using a primer pair that frames the D4R deletion (*Lane 5*). Negative controls were run without any template (*Lane 2*), with uninfected cDF-1 (*Lane 3*) cells and with the recombination plasmid pDM-Zgpt (*Lane 4*). Spike controls were performed with dMVA-ZG containing MVA-wt at 0.001-1% (*Lanes 6-9*). 1% corresponds to 1 000 pfu virus. A reaction with MVA-wt alone is shown on lane 10.

The absence of D4R containing wild type contaminants in the dMVA-ZG stock was also demonstrated by PCR. Primers were designed that bind to the viral genomic sequences flanking the D4R gene, resulting in a PCR product of 2430 bp in size when using MVA wt genomic DNA as the template. In order to avoid false positive signals resulting from viral sequences on the plasmid used for recombination, the chosen binding sites of the primers are located outside the VV sequences in the plasmid pDM-Zgpt (see Figure 1). With dMVA-ZG that contains the larger marker cassette in place of the D4R gene, a PCR fragment of 5624 bp was amplified (Figure 4). Due to the preferred amplification of smaller fragments in PCR reactions, down to 0.01% MVA-wt spiked into dMVA-ZG stock could be detected as an additional band (Figure 4, lane 7). No wt DNA was detected in the dMVA-ZG stock confirming therefore the purity of the defective virus.

### Generation of recombinant MVA by rescue of the D4R gene

For the generation of MVA recombinants with restored replication, we constructed the basic recombination plasmid pDM-D4R. This plasmid contains the D4R gene in its natural genomic context, with an additional multiple cloning site (MCS) for the insertion of genes of interest between the D4R and the D5R gene. Introduction of foreign genes into the D4R/D5R intergenic region was described earlier for recombinant vaccinia virus [29]. The genomic sequence adjacent to the D4R gene is highly conserved among vaccinia strains, suggesting that also in MVA, insertions in the D4R/D5R locus should not significantly interfere with virus viability. As proof of concept, we constructed the plasmid pDM-D4R-YF (Figure 1b) by placing an expression cassette for the yellow fever virus (YFV) surface proteins (prME) into the MCS of the basic recombination vector. Using this recombination plasmid together with the defective parental virus dMVA-ZG, we attempted the generation of a replication competent, YFV antigen-expressing MVA, termed rMVA-YF/D4. The design of the expression cassette containing the YFV prME sequence is described elsewhere [31].

Infection/transfection for homologous recombination between the defective MVA and the recombination plasmid pDM-D4R-YF and all subsequent steps were performed in the wt DF-1 cell line. Virus was harvested 2 days later and plated onto fresh cells under agarose overlay in the absence of selective agents. After 3 days, neutral red and X-Gal staining revealed clearly visible plaques of wild-type size. In this first round of plaque purification, already 70% of the plaques were no longer stainable by X-Gal, indicating the absence of parental β-galactosidase (β-Gal)-expressing dMVA-ZG virus. Twelve white plaques were randomly picked from this first round of plating, and were checked for absence of parental virus. Using cDF-1 cells that support the growth of both the parental dMVA-ZG and the D4R-restored rMVA-YF/D4 the isolates were first expanded in order to obtain sufficient material for quantitative tests. For phenotypic analysis, the isolates were subjected to plaque assay on cDF-1 cells. When plaques were stained with X-Gal to detect parental dMVA-ZG in the plaque assay, all 12 isolates were phenotypically free from β-Gal positive parental virus at a test sensitivity of approximately 1 parental virus among 50 plaques.

In order to further analyze the purity of the primary isolates, the isolates were subjected to analysis by quantitative PCR. As controls, DNA from uninfected cDF-1 cells and DNA from cDF-1 cells infected with wild-type MVA or with the parental dMVA-ZG were used. In the PCR, several genomic loci were targeted to identify the genotypes present in the isolates. Total genomic MVA DNA was quantified by use of a primer pair that targets an unrelated gene of the MVA genome-the MVA 173R (identical with VV B5R gene). In the rMVA-YF/D4 isolates 8.33-8.75 (mean 8.55) log genomic equivalents (GE)/ml of total genomic MVA DNA were detected (Table 1). A similar result (8.41) log GE/ml was obtained for the MVA wild-type control. The virus titer of the rMVA-YF/D4 isolates ranged from 2.68 × 10^6 ^to 1.20 × 10^7 ^TCID_50_/ml. From the copy number and the infectious titers, a normal copy-to-infectivity ratio of 117.6 (range 40-235.1) was calculated.

**Table 1:** Real-time PCR quantification of different target sequences in virus isolates obtained by the marker rescue procedure

Template	Total MVA^(1)^	YF prME^(1)^	lacZ^(1)^	AmpR^(1)^
rMVA-YF/D4 #1	8.54	8.44	3.33	3.83

rMVA-YF/D4 #2	8.38	8.25	3.17	4.02

rMVA-YF/D4 #3	8.54	8.30	3.47	4.02

rMVA-YF/D4 #4	8.58	8.24	3.37	4.07

rMVA-YF/D4 #5	8.69	8.42	3.37	3.89

rMVA-YF/D4 #6	8.33	8.14	3.45	4.08

rMVA-YF/D4 #7	8.64	8.49	3.66	4.11

rMVA-YF/D4 #8	8.54	8.50	3.61	4.02

rMVA-YF/D4 #9	8.74	8.55	3.48	3.97

rMVA-YF/D4 #10	8.39	8.27	3.54	4.07

rMVA-YF/D4 #11	8.75	8.49	3.42	3.96

rMVA-YF/D4 #12	8.46	8.44	3.56	4.06

Uninfected cDF-1	0.00	0.00	3.58	4.01

Wild-type MVA	8.41	2.62	3.27	3.93

dMVA-ZG	7.46	3.57	7.34	3.93

^(1)^Results are given in log_10 _genomic equivalents per ml, and are means of duplicate measurements

The presence of the gene of interest was measured by a YFV prME gene specific PCR reaction, where 8.14-8.55 GE/ml (mean 8.38) were found, consistent with the total MVA DNA quantification. Expectedly, only background signals (max. 3.57 log GE/ml) were obtained with the controls wild-type MVA and dMVA-ZG that did not contain any YFV sequences.

Two PCRs were performed specifically to detect intermediate structures in the recombinant samples. In the first analysis that targeted lacZ sequences from the parental virus dMVA-ZG, the isolates rMVA-YF/D4 ranged from 3.17 to 3.66 GE/ml which is equivalent to the background (3.58 log GE/ml) measured in uninfected DF-1 cells. A second analysis targeted the ampicillin resistance gene to detect intermediate viral genomes that form during recombination and that still contain the complete plasmid backbone. Also in this analysis, the isolates gave results at the background level of the controls, suggesting that residual intermediate structures were practically absent in the isolates.

### The MVA intergenic D4R/D5R insertion locus

**Figure 5:**
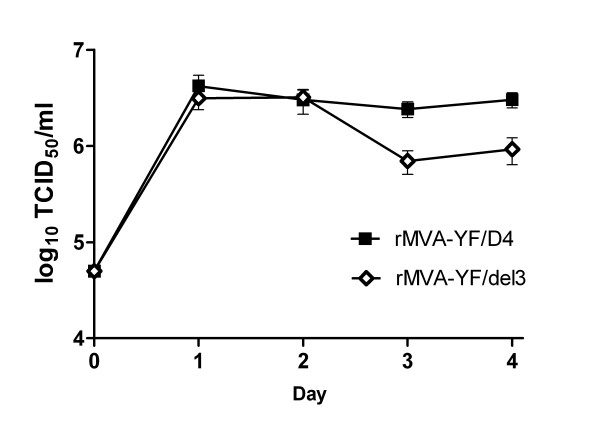
**Growth kinetics of the recombinants rMVA-YF/D4 and rMVA-YF/del3**. DF-1 cells were infected with rMVA-YF/D4 and rMVA-YF/del3 at moi = 0.05. Cells were harvested at the indicated time points and the viral titers were determined by TCID_50 _titration. Mean values ± SEM are shown (*n *= 6).

In the present approach, a foreign gene was inserted into the virus genome downstream of the essential D4R gene and upstream of the putative promoter of the D5R gene, as described previously for replicating vaccinia strains. Commonly used insertion sites for MVA recombinants include the HA gene which is supposed to be silent in MVA, and the genomic deletion III (del3) region [18,40]. For MVA, insertion into the D4R/D5R intergenic locus has not been described so far. In order to assess whether manipulation of this locus affects the viability of the multifold attenuated MVA, the growth of the new recombinant MVA was compared to a conventional construct. For this purpose, growth experiments were performed in parallel with rMVA-YF/D4, and with a conventionally generated recombinant MVA rMVA-YF/del3 that contains the analogous foreign sequences in the del3 locus [31]. With rMVA-YF/D4 that carries the foreign gene in the D4R/D5R locus, growth rates and maximal titers were at least as high as with the control construct where the del3 region was used for insertion (Figure 5), indicating that insertion of foreign DNA between the MVA D4R and D5R genes does not interfere with virus growth.

### Expression of a gene of interest without clonal selection

**Figure 6:**
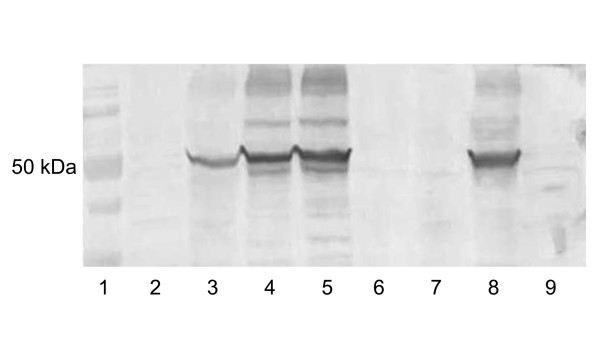
**YFV antigen expression from a non-purified virus stock**. Western blot analysis of yellow fever prME protein expression was performed at different passages of an unselected rMVA-YF/D4 stock, using a polyclonal anti YFV guinea pig serum that detects the YFV E protein as a ~50 kDa band. The blot shows the primary virus crude stock following infection of DF-1 cells with dMVA-ZG and transfection with pDM-D4R/YF (*lane 2*), and harvest from the first (*lane 3*), the second (*lane 4*) and the third passage (*lane 5*) of this stock. In parallel, the plasmid pd3-YFprMEco that lacks the D4R gene was transfected into dMVA-ZG infected DF-1 cells (*lane 6*) and passaged once (*lane 7*). As a positive control, clonally purified rMVA-YF/del3 was used (*lane 8*). Uninfected cells are shown on lane 9.

In conventional selection procedures using non-viral marker genes and chemicals for selection, clonal purification of the recombinant virus is crucial to obtain pure stocks, because the recombinants are rapidly overgrown by residual parental virus, once the selective pressure is removed. In contrast, the dominant host range selection by rescue of the MVA D4R gene provides a constant growth advantage to the recombinant over the non-replicating parental virus. To investigate whether efficient transgene expression could be achieved also without any plaque purifications, DF-1 cells were infected with dMVA-ZG and transfected with the rescue plasmid pDM-D4R/YF, and the resulting harvest was serially passaged three times in DF-1 cells without subcloning. For passaging, infections were harvested after 4 days. Crude stocks were prepared, diluted 1:5, and used in a new round of virus propagation. From each passage, cell lysates were prepared, and the YFV prME expression levels were analyzed by Western blotting (Figure 6).

The YFV envelope protein was detectable as a 50 kDa band already after one virus passage (lane 3). After two passages (lane 4), expression was similar to the control rMVA-YF/del3 that had been purified by conventional clonal selection (lane 8). When the same procedure was done with a control plasmid that contained the YFV prME cassette but not the D4R gene as a selectable marker, E protein expression was undetectable at all passages (lanes 6, 7). This indicated that the transgene expression from the transfected plasmid DNA itself was neglectable, and that the YFV E protein detected by Western blotting reflected replicating recombinant MVA virus.

In order to investigate the proportion of recombinant virus into more detail, the virus from the different passages was subjected to plaque assay on cDF-1 cells that equally support growth of the replicating, i.e. recombinant and the defective parental MVA. Plaques were immune-stained with antisera directed either to yellow fever virus prME antigen or to vaccinia virus, thereby discriminating recombinant prME-expressing plaques from non-expressing MVA plaques.

The percentage of prME-expressing rMVA-YF/D4 plaques was 46% already after the initial recombination, reaching a plateau after two passages at about 98% (Table 2).

**Table 2:** Percentage of transgene expressing virus plaques after passages without clonal selection

Passage	prME Positive Plaques (%)
Primary crude stock	46.3 ± 1.33^1^

First passage	92.1 ± 0.23^1^

Second passage	98.1 ± 0.78^1^

Third passage	97.1 ± 2.86^1^

Purified rMVA-YF/del3	99.2^2^

1) Mean of two experiments ± SD

2) One experiment

## Discussion

This study describes a method to create recombinant MVA vectors, based on the principle of dominant host range selection upon rescue of the viral D4R gene [29]. The presented approach allows for rapid isolation of the target recombinant, resulting in parental virus-free recombinant stocks in a single round of plaque purification. While generation of recombinants of the highly attenuated MVA is traditionally more difficult than of fully replicating vaccinia viruses, the D4R-dominant host range selection represents a simple and stringent alternative to integrate genes of interest into MVA. Traditional selection methods frequently involve the introduction of foreign selection marker genes, which may be undesired for the use in live vaccines. Marker genes may be de-stabilized by flanking tandem DNA repeats [18,41], which however demands for additional plaque purifications to select for the marker-free genotype. As an alternative to the homologous recombination method to generate recombinant vaccinia virus, the cloning and manipulation of the entire vaccinia genome in a bacterial artificial chromosome (BAC) has been described [42]. Recently, this method was also applied to MVA, and was proposed as a rapid approach for the generation of recombinant MVA [43,44]. While the BAC technology offers the advantage of targeting any genomic locus of MVA, the method is dependent on the use of a helper virus, and results in recombinants that carry the complete BAC plasmid backbone in addition to the gene of interest. The BAC construct can also be designed to favour its excision by spontaneous homologous recombination [44], requiring however for additional plaque purifications or serial passage similar to the previously described marker destabilization. In contrast, the dominant host range selection procedure directly results in recombinant viruses without any foreign marker sequences by re-introduction of the essential viral D4R gene as the selection marker.

As a complementing cell line for the defective MVA, we generated a D4R-expressing avian DF-1 cell line, because of the advantageous properties of these cells with respect to virus propagation and plaque formation. Our approach of engineering the D4R gene into the cells by retroviral transduction has been applied previously to a simian cell line [34], and now successfully to an avian cell line, suggesting that this strategy is applicable to further cell lines that may be considered for propagation of MVA. In the present approach, the complementing DF-1 cell line supported growth of the defective MVA to levels similar to the wt virus. A D4R complementing DF-1 cell line by Garber and colleagues [27] who used a different technique resulted in similar defective MVA titers, i.e. approximately 5 × 10^7 ^pfu/ml at 24 hrs post infection. For use in the dominant selection procedure, the absolute titer of the defective virus is not critical. However, the growth kinetics of dMVA suggest that the expression level of the vaccinia uracil DNA glycosylase by the cell line cDF-1 was not limiting for viral replication, hence providing full complementation of the defective virus.

The approach of selecting recombinant virus by reconstitution of the viral D4R gene allowed isolation of clonal recombinant virus in a single round of plaque purification. The parental defective MVA contains a lacZ marker cassette that supports screening of descendant recombinant virus. In the initial plating, about 30% of the plaques appeared blue, indicating that they still contained parental virus. However, by picking white plaques, all of the 12 isolates were found to be already pure recombinants without intermediate structures as analyzed by quantitative PCR.

Because of the constant selection pressure against the parental defective virus, it is feasible to obtain and amplify recombinant virus stocks directly after infection/transfection without any further purification steps. This simplified method is no option for the production of well-defined virus stocks, but it represents a powerful tool e.g. if rapid and efficient transgene expression for analytical purposes is desired. As an example, we generated a recombinant virus stock without any plaque purification, achieving 98% purity in only two blind passages. The low amount of residual non-expressing virus suggests that this method is also useful if quantitative assessment of expression levels, e.g. to perform promoter studies. Principally, this type of enrichment procedure is feasible with any stringent selection marker. This has been demonstrated previously with the MVA F13L gene as a selectable plaque size marker [24], where close to 100% recombinant virus was achieved after 5 blind passages. However, enrichment procedures that make use of non-essential genes such as F13L provide a less stringent selection pressure, because the parental virus is still replicating, though at a reduced growth rate. On the other hand, deletion of non-essential host range genes such as K1L provides selective conditions in certain cell systems [18], but growth restriction of the parental virus is not maintained once the recombinant virus stock is transferred to avian cells-the normal substrate for MVA. While recombinant stocks obtained in this way are bound to be overgrown by parental virus under standard culture conditions, the defect in D4R-deleted MVA provides constant selective pressure in favor of the D4R bearing recombinant virus in any natural host.

## Conclusions

The D4R dominant host range selection represents a quick and stringent method to generate recombinant MVA viruses. The method represents an alternative to current selection techniques that rely on additional marker genes, chemical agents, and multiple rounds of plaque purification. In contrast to traditional homologous recombination protocols and to the recently described BAC cloning procedure, the less laborious D4R-dominant host range selection approach by principle does not require foreign marker genes, resulting in MVA recombinants that are free from marker gene sequences and that thus meet the state-of-the-art requirements for use as live vaccines.

## Abbreviations

MVA: Modified vaccinia virus Ankara; CEF: Chicken embryo fibroblast cells; VV: Vaccinia virus; PNK: RNA-activated protein kinase; wt: Wild-type; NEAA: Non-essential amino acids; YFV: Yellow fever virus; prME: Precurser protein of the matrix and envelope proteins; TCID_50_: Tissue culture infectious dose 50%; pfu: Plaque forming unit; MCS: Multiple cloning site; β-Gal: E. coli β-Galactosidase; GE: Genomic equivalents; del3: MVA genomic deletion III locus.

## Competing interests

G.H., B.S., F.G.F., and T.R.K. are employees of Baxter. G.H., B.S., F.G.F., and T.R.K. report having an equity interest in Baxter. P.S.R. no competing interests.

## Authors' contributions

PSR performed the experiments including the generation of the complementing cell line and the recombinant viruses, and drafted the manuscript. BS generated the basic YFV constructs, and contributed to the design of the viruses and to data analysis. TRK supervised design and manuscript writing. FGF conceived the study and contributed to manuscript writing. GH and BS coordinated the experiments and GH finalized the manuscript. All authors have critically read the final manuscript.
